# Assessing the Impact of an Indigenous Palliative Care Nurse Navigator: A Program Evaluation

**DOI:** 10.1177/26892820251385777

**Published:** 2025-10-15

**Authors:** Iain John McInnis, Cara Alison Bablitz, Juliet Foster, Trevor Morey, Natasha Gougeon, Reuben Quinn

**Affiliations:** ^1^ University of Alberta College of Natural and Applied Sciences, Edmonton, Canada.; ^2^ University of Alberta Faculty of Medicine & Dentistry, Edmonton, Canada.; ^3^ Alberta Health Services, Edmonton, Canada.; ^4^ Inner City Health Associates, Toronto, Canada.; ^5^ Indigenous Wellness Clinic, Royal Alexandra Hospital, Edmonton, Canada.; ^6^ Faculty of Arts, University of Alberta, Edmonton, Canada.

**Keywords:** culturally safe care, health equity, Indigenous health, nurse navigator, palliative navigation, underserved populations

## Abstract

**Introduction:**

Indigenous peoples in Canada face significant health disparities rooted in colonization and experience inequitable access to palliative care. Patient navigation has proven effective in both Indigenous and palliative contexts, but integration of these roles has not been studied. This evaluation defines the scope of a patient navigator specializing in palliative care for Indigenous peoples and explores how it addresses service gaps.

**Methods:**

A program evaluation tool was developed to track key performance indicators (KPIs) of an Indigenous Palliative Care Nurse Navigator (IPCNN) over six months. Responsibilities were organized into three domains: (1) social vulnerabilities, (2) mainstream palliative care navigation, and (3) barriers to palliative care for Indigenous peoples. Sixteen categories further captured the scope of responsibilities. KPIs were classified as either “working” indicators, reflecting activities undertaken for future outcomes, or “outcome” indicators, which tracked the achievement of results. A single IPCNN provided care to 164 Indigenous patients across Alberta, Canada with a prognosis of less than 24 months, conducting initial assessments and maintaining ongoing contact through home visits, clinic appointments, and phone calls every two to four weeks. KPIs were tabulated and analyzed by frequency (low, moderate, and high), domain, category, and classification.

**Results:**

One IPCNN logged 714 activities across three domains: social vulnerabilities (191), mainstream palliative care navigation (394), and barriers to palliative care for Indigenous peoples (129). Most KPIs (77%) were “working” activities, reflecting upstream tasks required to achieve outcomes. The majority of KPIs addressing barriers to palliative care for Indigenous peoples were high-frequency activities.

**Discussion:**

The IPCNN role supports Indigenous patients to navigate gaps in palliative care delivery, including distrust of health care providers, jurisdictional challenges, and incongruencies between Indigenous culture and Western medicine. These findings underscore the potential of Indigenous palliative care navigation as an effective intervention to improve the accessibility and quality of palliative care for Indigenous peoples.

## Background

Significant health disparities exist between Indigenous and non-Indigenous populations in Canada. As a result, Indigenous peoples in Canada have a life expectancy consistently shorter than non-Indigenous peoples, with some groups experiencing a gap of up to 10 years.^1^ The factors that contribute to this disparity are complex and interrelated—the historical context of colonization, residential schools, and resulting intergenerational trauma have caused significant disadvantages in many social determinants of health and result in much lower life expectancies among Indigenous peoples in Canada.^2^ Indigenous peoples consistently report lower on measures of income, education, employment, and housing in comparison to non-Indigenous Canadians.^3^ This disparity contributes to an increased risk of illness and death among Indigenous peoples, highlighting a heightened need for culturally safer end-of-life care. Unsurprisingly, Indigenous peoples also suffer from inequitable access to palliative care due to systemic and individual-level barriers.^4,5^ Because of this, Indigenous Peoples gain significant benefits from culturally safer professional navigation of Canada’s complex and disjointed medical and social service systems at the end of life.

Navigating the health care system unassisted can be an overwhelming experience, especially for individuals facing multiple barriers to care. Patient navigation is a “strategy for overcoming patient-level and system-level barriers … to improve access to and coordination of timely care for those most in need.”^6^ Improved access is accomplished with the implementation of a patient navigator—a health care professional that specializes in case management, coordination of care, managing health care transitions, and reducing barriers to care. Patient navigators can vary in their background and training—patient navigation programs may recruit nurses, social workers, community workers, or peer navigators, depending on the scope and responsibilities of the role.^7^ In the present study, navigation was provided by a registered nurse. Multiple studies have demonstrated that patient navigation is a cost-effective intervention that significantly improves patient outcomes and is an effective way to address health disparities experienced by underserved populations.^8,9^

There is growing recognition that patient navigation is particularly important in palliative care. Palliative care navigation has been shown to ease common challenges near the end of life, such as symptom relief, quality of care, communication efficacy, advanced care planning, achieving prognostic alignment, transitions of care, and bereavement care for families.^10,11^ Furthermore, patient navigation has been shown to be effective in overcoming barriers to care experienced by Indigenous peoples. In addition to the conventional responsibilities of a patient navigator, Indigenous patient navigators address challenges specific to this population, such as jurisdictional issues, racism within the health care system, patient mistrust of health care providers and the health care system, and attaining cultural support.^12^ However, despite early evidence demonstrating the effectiveness of both palliative and Indigenous navigation, to our knowledge, there is no research investigating the potential for Indigenous-specific palliative care navigation to facilitate equitable access to palliative care among Indigenous populations. Additionally, research investigating patient navigation has primarily focused on assessing its efficacy by measuring differences in health outcomes, with minimal process-oriented research examining how navigators spend their time and on which specific tasks.^13^ This research contributes to addressing this dearth in the literature by conducting systematic research evaluating key performance indicators (KPIs) of an Indigenous Palliative Care Nurse Navigator (IPCNN).

The Palliative Care Outreach and Advocacy Team (PCOAT) is a program based in the Indigenous Wellness Clinic at the Royal Alexandra Hospital on Treaty 6 Territory in Edmonton, Alberta, Canada, that provides palliative care to structurally vulnerable individuals. Patients of the program pay no fee for services as Canada’s health care system provides free access to medical services through provincially regulated public health insurance plans. The inclusion of the Medicine Chest Clause in the signing of Treaty 6 has been historically viewed by many First Nations as affirming a treaty right to health, underscoring the importance of accessible and culturally safer health care services for Indigenous peoples of this region.

In 2022, through the Government of Alberta’s Palliative and End-of-Life Care Grant Fund, the PCOAT program received funding to establish an IPCNN role to provide Indigenous patients with a life-limiting illness navigation of the medical and social service systems, and overcome barriers to palliative care unique to Indigenous peoples. A registered nurse with over 10 years of experience in palliative care was hired for this position to ensure that the navigator had specialized knowledge in providing a palliative approach to care. The patient-facing activities of the IPCNN were broad in scope, but generally involved conducting an initial assessment with each patient and providing ongoing support through home visits, clinic appointments, and telephone contact approximately every two to four weeks, with frequency tailored to patient needs. Additionally, the scope of the IPCNN built upon and extended the important work of an interdisciplinary palliative care team, encompassing navigation tasks such as identifying and addressing individual barriers to care (housing instability, transportation, and financial constraints), assisting patients in accessing social services, coordinating care across multiple care providers, and advocating for patients in complex institutional and community contexts.^6,7^

With the addition of the IPCNN, the dedicated staff of the PCOAT program includes an Indigenous palliative care specialist physician and a registered nurse at 0.6 and 0.8 full-time equivalents, respectively. By developing a delivery model for palliative care that is primarily informed by community members, the PCOAT team provides patient-centered and equitable community-based palliative care. The team’s palliative care physician is available to patients from 9:00 a.m. to 5:00 p.m. three days a week and is on call 24/7; the registered nurse is available to patients from 9:00 a.m. to 5:00 p.m. from Monday to Thursday. Referrals are low barrier and can be input by Alberta Health Services’ health care providers through the electronic medical record or received from others such as community members or patient supports through phone or email. The IPCNN on the PCOAT team received 164 patient referrals and 445 patient encounters including patient outreach, telephone calls, documentation, and clinical support over the six-month data collection period.

### Objective

The purpose of this program evaluation was to describe the scope of activities performed by an IPCNN and to assess how this role may help address gaps in the delivery of community-based palliative care for Indigenous peoples in Alberta, Canada. Specifically, the evaluation examined the types and distribution of activities carried out by the IPCNN and considered how these activities support access to palliative care and social services by addressing common barriers experienced by Indigenous individuals with life-limiting illnesses.

### Design

To illustrate the various responsibilities of an IPCNN, the authors developed an original program evaluation tool modeled after the work done by Robinson et al.^11^ in their evaluation of a patient navigator position within the Palliative Education and Care for the Homeless (PEACH) program in Toronto, ON. In consultation with members of the PEACH team, we developed KPIs addressing three domains: (1) addressing social vulnerabilities, (2) mainstream palliative care navigation, and (3) addressing barriers to palliative care for Indigenous Peoples. The KPIs in domains one and two were largely derived from responsibilities of a patient navigator described in the PEACH program’s study plus descriptions of the roles of nurse navigation.^14,15^ Domain three KPIs were created based on current literature related to general Indigenous patient navigation and Indigenous palliative care needs as there is a lack of research examining Indigenous palliative care navigation.^12,16–18^ A total of 16 KPI categories and 67 individual KPIs were included in the program evaluation tool. Additionally, KPIs were categorized as either “working” indicators, reflecting activities undertaken in service of future outcomes, or “outcome” indicators, which tracked the achievement of desired results.

### Setting/Subjects

The IPCNN position had a province-wide scope, as their case load included patients from across Alberta; however, most patients were members of Edmonton’s inner-city community. The IPCNN position was occupied by one individual throughout the duration of the study. Throughout the study, the IPCNN supported approximately 40 patients at a time with minor fluctuations in numbers based on frequency of deaths and referrals. To become an IPCNN patient, the individual must have had a prognosis of less than 24 months and self-identify as Indigenous. This study was reviewed by the University of Alberta’s Research Ethics Office, which granted a waiver of ethics review as it met the conditions for exemption under the Tri-Council Policy Statement: Ethical Conduct for Research Involving Humans (TCPS 2).

### Measurements

KPIs were tracked by the IPCNN for six months, from April to October 2024. When the IPCNN completed an activity or obtained an outcome that corresponded to a specific KPI, it was recorded in an anonymized Excel spreadsheet for tabulation. To the best of our knowledge, the PCOAT’s IPCNN role is the first of its kind, meaning there was no literature from which to build a complete catalog of KPIs capturing all activities related to Indigenous palliative care navigation. Because of this, some activities completed by the IPCNN during the data collection period were not anticipated when designing the tracking document. To account for this, the tracking document was edited to include unexpected KPIs as they occurred. The activities of the IPCNN were also often documented in the patient’s electronic medical record, depending on the type of activity and/or outcome.

### Data analysis

The total number of occurrences for each individual KPI were tabulated to calculate the frequency of each KPI across a six-month period. Additionally, the number of occurrences were calculated for the 16 KPI categories, the three KPI domains, “working” versus “outcome” KPIs, and the aggregate total of all KPIs to assess their respective frequencies. Furthermore, individual KPIs were categorized by their respective frequencies into low frequency (0–1 occurrences), moderate frequency (2–9 occurrences), and high frequency (10+ occurrences).

Collated data were then analyzed by comparing KPIs frequencies across the three domains, 16 categories, and the “working” versus “outcome” classifications. Cross-tabulation tables were used to assess frequency levels (low, moderate, and high) within each domain and category (Table 2), and to compare “working” versus “outcome” indicators across domains and the aggregate total (Table 3). This analysis described the type and distribution of work performed by the IPCNN.

## Results

Between April and October 2024, the IPCNN logged an aggregate total of 714 activities captured by the KPIs characterizing the work required of this role. Activities across each of the three KPI domains showed moderate variation in their respective frequencies: 394 events recorded in mainstream palliative care, 191 events recorded in addressing social vulnerabilities, and 129 events recorded in addressing barriers to palliative care for Indigenous peoples (Table 3). The 16 KPI categories demonstrated much more variation, with some recording as little as zero events and others surpassing 100 events such as referrals and coordination, and health care (Table 2).

Individual KPIs that the navigator recorded most frequently included coordinating with Nation health services (16), medication recommendations to a primary care provider (16), collaborating/networking with health care providers (16), improved symptom management (17), Noninsured health benefits application and navigation (18), relationship building with Indigenous communities (21), home visits (22), coordinating homecare (29), coordinating appointment reminders (32), identifying new patients (39), providing health screening/assessments (40), providing emotional/social support (40), patient care coordination (rounds) (40), coordinating appointment scheduling (49), and chart review (73) (Table 1).

**Table 1.** Individual and Domain Totals for KPI Frequency

	KPI frequency
Domain 1: Addressing Social Vulnerabilities	
Category 1: Housing	
KPI 1.1 Submit housing application	**1**
KPI 1.2 Coordinate housing application	**6**
KPI 1.3 Housing application advocacy	**1**
KPI 1.4 Accompany to view housing	**0**
KPI 1.5 Housing secured	**5**
KPI 1.6 Arrange furniture	**0**
Category 2: Income	
KPI 2.1 Coordinated AISH application	**6**
KPI 2.2 Secured AISH	**2**
KPI 2.3 Coordinated application for other income support	**4**
KPI 2.4 Other income support secured	**5**
Category 3: Food (In) Security	
KPI 3.1 Connect to food bank	**3**
KPI 3.2 Provide snacks/food during clinic appointments	**13**
KPI 3.3 Coordinate other types of food insecurity support	**2**
Category 4: Referrals and Coordination	
KPI 4.1 Coordinated homecare	**29**
KPI 4.2 Coordinated medication delivery/pick up	**10**
KPI 4.3 Coordinated ACP discussion with family	**9**
KPI 4.4 Coordinated patient safety planning	**1**
KPI 4.5 Coordinated taxi/transit cards/other travel	**6**
KPI 4.6 Coordinated appointment scheduling	**49**
KPI 4.7 Coordinated appointment reminders	**32**
KPI 4.8 Referral for grief support	**5**
KPI 4.9 Arranging referrals (other)	**2**
Domain 1 total:	**191**
Domain 2: Mainstream Palliative Care Navigation	
Category 5: Administrative Navigation	
KPI 5.1 Completing paperwork for programs	**1**
KPI 5.2 Insurance application-Palliative Blue Cross	**8**
KPI 5.3 Insurance secured	**3**
KPI 5.4 Facilitate personal directive/power of attorney	**2**
Category 6: Accompaniment to Medical Appointment	
KPI 6.1 Attended appointment	**3**
KPI 6.2 Accompaniment to palliative care unit admission	**0**
KPI 6.3 Facilitate/attend telemedicine appointment	**0**
KPI 6.4 Visit patient in hospital and advocate for supportive care	**6**
Category 7: Access to Interdisciplinary Care	
KPI 7.1 Connect to dentist/ophthalmologist/audiologist	**2**
KPI 7.2 Connect to occupational/physical therapy	**6**
KPI 7.3 Connect to addiction worker/intensive care manager	**6**
KPI 7.4 Connect to psychologist/psychiatrist	**1**
KPI 7.5 Aided in referral to specialist	**14**
Category 8: Psychosocial Support	
KPI 8.1 Provided emotional/social support to patient	**40**
Category 9: Capacity Building/Partnership Development	
KPI 9.1 Deliver community workshops	**0**
KPI 9.2 Conducted presentation of palliative care/IPCNN role	**4**
KPI 9.3 Involvement in media representations of PCOAT/IPCNN	**0**
KPI 9.4 Collaborate/networking with health care providers	**16**
Category 10: Health Care	
KPI 10.1 Follow up on tests/results	**6**
KPI 10.2 Dressing changes	**6**
KPI 10.3 Chart review (patient monitoring)	**73**
KPI 10.4 Patient care coordination (rounds)	**40**
KPI 10.5 Improved symptom management	**17**
KPI 10.6 Medication recommendations to PCP	**16**
KPI 10.7 Home visit	**22**
KPI 10.8 Provide health screening/assessment	**40**
KPI 10.9 Advanced care planning (goals of care)	**4**
KPI 10.10 Obtain speciality services/devices	**2**
Category 11: Patient Outreach and Advocacy	
KPI 11.1 Identify new patients for PCOAT/IPCNN	**39**
KPI 11.2 Optimized patient care-attended case conference meeting	**3**
KPI 11.3 Facilitate pain management conversation	**13**
KPI 11.4 Facilitate patient care contract	**1**
Domain 2 total:	**394**
Domain 3: Addressing Barriers to Palliative Care for Indigenous Peoples	
Category 12: Indigenous Food Insecurity Programs	
KPI 12.1 Coordinate Jordan’s principle funding for food	**0**
Category 13: Indigenous Referrals and Coordination	
KPI 13.1 Coordinating interpretation services for appointments	**0**
KPI 13.2 Coordinating relationship building/connection with indigenous communities	**21**
KPI 13.3 Referral for cultural support	**10**
Category 14: Building Relationships with Distrusting Indigenous Patients	
KPI 14.1 Established trust and genuine relationship with patient	**29**
Category 15: Capacity Building and Partnership Development with Indigenous Communities	
KPI 15.1 Address jurisdictional barriers	**17**
KPI 15.2 Coordinating with Nation health services	**16**
KPI 15.3 Networking with indigenous community leaders	**4**
KPI 15.4 Education on cultural practice to health care providers	**9**
KPI 15.5 Providing antiracism education to health care providers	**4**
KPI 15.6 General education activities re indigenous culture and history	**1**
Category 16: Indigenous Administrative Navigation	
KPI 16.1 Non-Insured Health Benefits (NIHB) application and navigation	**18**
KPI 16.2 Assisting with administrative tasks re land issues	**0**
Domain 3 Total:	**129**

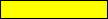
 Working KPI.

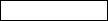
 Outcome KPI.

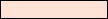
 Low frequency (0–1).

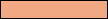
 Moderate frequency (2–9).

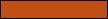
 High frequency (10+).

ACP, advanced care planning; AISH, assured income for the severely handicapped; IPCNN, Indigenous palliative care nurse navigator; KPIs, key performance indicators; PCOAT, Palliative Care Outreach and Advocacy Team; PCP, primary care provider.

**Table 2. KPI Frequency (Low, Moderate, or High) by Category:** 

		Number of KPIs per frequency category			
KPI category	Total number of events	High	Moderate	Low	
Domain 1: Addressing Social Vulnerabilities					
Housing	13	**0**	**2**	**4**	
Income and related supports	17	**0**	**4**	**0**	
Food (In)security	18	**1**	**2**	**0**	
Referrals and coordination	143	**4**	**4**	**1**	
Total	**191**				
Domain 2: Mainstream Palliative Care Navigation					
Administrative navigation	14	**0**	**3**	**2**	
Accompaniment to medical appointment	9	**0**	**2**	**2**	
Access to interdisciplinary care	29	**1**	**3**	**1**	
Psychosocial support	40	**1**	**0**	**0**	
Capacity building and partnership development	20	**1**	**1**	**2**	
Health care	226	**6**	**4**	**0**	
Patient outreach and advocacy	56	**2**	**1**	**1**	
Total	**394**				
Domain 3: Addressing Barriers to Palliative Care For Indigenous Peoples					
Indigenous food insecurity programs	0	**0**	**0**	**1**	
Indigenous referrals and coordination	31	**2**	**0**	**1**	
Building relationships with distrusting Indigenous patients	29	**1**	**0**	**0**	
Capacity building and partnership development with Indigenous communities	51	**2**	**3**	**1**	
Indigenous administrative navigation	18	**1**	**0**	**1**	
Total	**129**				

**Table 3. KPI Frequency: Working versus Outcome:** 

		Number of KPIs by measurement type	
Domain group	Total number of events per domain	Working	Outcome
Domain 1: Addressing Social Vulnerabilities	191	121 (63%)	70 (37%)
Domain 2: Mainstream Palliative Care Navigation	394	345 (88%)	49 (12%)
Domain 3: Addressing Barriers to Palliative Care for Indigenous Peoples	129	83 (64%)	46 (36%)
Aggregate KPI total	714	549 (77%)	165 (23%)

The IPCNN had 226 occurrences of health care KPIs, resulting in direct in-person patient care, including dressing changes (6), home visits (22), and providing health screening/assessments (40). Addressing the social needs of Indigenous patients with a life-limiting illness was found to be a key responsibility of the IPCNN, with 191 individual events logged within this domain (Table 2). Of those, the majority involved referrals and coordination with 143 events. Seventy-seven percent of documented KPIs were classified as working activities, reflecting the upstream tasks required to achieve outcomes (Table 3).

Domain three, which addresses navigation areas for Indigenous peoples, was primarily categorized as moderate (2–9 occurrences) and high frequency (10+ occurrences) activities (Table 2); Nine of the individual KPIs in domain three were categorized as moderate or high frequency, while only four activities were categorized as low frequency (Table 2). Activities that supported culturally safer care include facilitating referrals for cultural support (10), educating health care providers on cultural practices (9), providing anti-racism education to health care providers (4), and networking with Indigenous community leaders/elders regarding palliative care (4) (Table 1). The IPCNN also completed activities associated with overcoming systemic barriers at a high frequency, recording 16 activities of coordinating with Nation health services and 17 of facilitating the resolution of jurisdictional issues (Table 1).

## Discussion

This study characterizes the activities undertaken by an IPCNN and the relative frequency of these activities to determine how this service may assist in delivering community-based palliative care to Indigenous patients. It adds to a growing body of literature on both palliative and Indigenous navigation as independent interventions to improve health outcomes, while addressing a gap in the literature by characterizing a role that combines these interventions into a single patient navigation position. By outlining the type and distribution of activities performed by the IPCNN, this analysis highlights how the role responds to unmet needs and may help fill gaps in existing palliative care service delivery for Indigenous peoples in Alberta. This is an important contribution to the literature given persistent inequities in palliative care access for Indigenous peoples,^4,5^ and it offers a framework that community-based palliative care teams serving Indigenous patients can use to assess and track the activities of patient navigators using the KPIs set out in this study.

Research demonstrates that health inequities experienced by Indigenous peoples are the result of disadvantages across all social determinants of health stemming from the lasting effects of colonization.^3^ Addressing social determinants of health for structurally vulnerable individuals is essential to improve access to palliative care—strategies to accomplish this include utilizing harm reduction approaches and ensuring better integration of social services and health care services.^11,19,20^ Consistent with these insights, this evaluation found that addressing the social needs of Indigenous patients with a life-limiting illness was a key responsibility of the IPCNN (191 events). Referrals and coordination accounted for the majority of this total (143 events), highlighting the need for structurally vulnerable populations to receive support in navigating Canada’s fragmented medical and social service systems.^21^

Supporting the results of Robinson et al.,^11^ this research found that the majority of logged KPI events were “working” activities (77%) in comparison to “outcome” activities (Table 3). In this context, working activities describe tasks that are completed in the service of a downstream outcome. Organizing the data in this way enables an analysis of the hidden work undertaken by an IPCNN, whether it goes unrealized (though it may still yield benefits such as building rapport with patients), or does not produce immediate results. For example, eight activities related to housing advocacy were required to secure five successful housing applications (Table 1), which further facilitate downstream outcome KPIs such as home care (29) and medication delivery (10). This highlights the disproportionate effort required to achieve a single favorable outcome within this role. However, even one favorable outcome—such as securing stable housing—can play a pivotal role in an individual’s ability to access palliative care.

Another important finding was that health care-related activities were all moderate or high-frequency activities. This differs from other patient navigator roles, as many are occupied by social workers, community workers, or lay persons.^7^ We argue that the frequent face-to-face interaction necessary for health care activities played an important role in the ability of the IPCNN to build meaningful patient/navigator relationships, establish trust, and ultimately act as a key link between patients and the larger health care system.

KPIs belonging to domain three, which address barriers to care unique to Indigenous peoples, were primarily categorized as moderate (2–9 occurrences) and high frequency (10+ occurrences) activities (Table 2). This indicates that, relative to other activities, addressing barriers to palliative care for Indigenous Peoples was a significant responsibility of this role and underscores the need for a role dedicated to supporting Indigenous peoples in overcoming the unique barriers faced in accessing palliative care.

One of the primary individual-level barriers to care experienced by Indigenous peoples is mistrust of the medical system and health care professionals due to previous experiences of perceived racism, discrimination, and poor quality of care.^22^ Because of this, one of the main tasks undertaken by the IPCNN was to establish trust with Indigenous patients and communities. By employing a patient-centered, trauma-informed, harm reduction approach and establishing rapport through a sustained commitment to supporting individual patients’ care, the IPCNN had 29 occurrences of building meaningful trust/relationships (Table 1). The patient-navigator relationship has been identified as a key factor in enabling navigators to provide effective emotional support,^23^ which patients frequently cite as one of the most valuable services offered by navigators.^24^ Additionally, trust between patient and health care provider has been shown to positively influence patient management outcomes, especially in the treatment of chronic disease,^25^ which is prevalent among this patient demographic.

Fundamental incongruencies between Indigenous culture and Western medicine in palliative care represent another barrier that Indigenous peoples face at the end of life. Receiving care in a hospice out of community and/or dying in hospital is often incompatible with traditional values regarding family and community. Furthermore, traditional cultural practices such as smudging ceremonies are often difficult to accommodate in institutional settings.^16^ KPIs that assist in overcoming these incongruencies took place at a moderate (2–9 occurrences) to high (10+ occurrences) rate, indicating that addressing barriers to culturally safer palliative care was one of the focal responsibilities of the IPCNN (Table 1). This is critical, as culturally safer care improves patient satisfaction, patient trust, and adherence to treatment,^26^ allowing for a peaceful and dignified death.

Systemic barriers to care are issues that arise from the health care system itself, such as its geographic placement, organizational structure, and legislative mandate. For example, in rural Indigenous communities, health care services are often scarce due to geographic isolation, language barriers and financial barriers.^27,28^ Furthermore, Canada’s fragmented health care system results in issues concerning continuity of care, especially for Indigenous individuals who must often circumnavigate jurisdictional challenges tied to the division of responsibility between federal and provincial governments regarding the delivery of health care services. For example, provincial palliative care services may not be provided on a Nation due to health care delivery falling under federal jurisdiction.^4,27^ The IPCNN frequently undertook tasks to overcome these barriers, such as coordinating with Nation health services (16 events) to assist with the repatriation of patients to their home communities to die in their chosen location (Table 1). These high-frequency activities underscore the need for dedicated resources to support navigation of systemic barriers to ensure equitable access to palliative care for all Indigenous peoples.

### Limitations

This study has potential limitations. As there was no control group to compare against, we are unable to conclusively assume that the IPCNN achieved a greater number of favorable outcome activities relative to patients without an IPCNN supporting their care. Additionally, the findings presented here may not be generalizable to other populations due to differences in local health care and social service policy, and the diversity of Indigenous communities and their respective resources. Finally, time constraints required that data collection occur across a six-month period—this relatively short data collection period may affect the representativeness of the collected data and may prevent some meaningful outcomes from being captured, leading to an underrepresentation of outcome KPIs being achieved during the study period.

## Conclusions

This study confirms the hypothesized need for Indigenous palliative care navigation, as it demonstrates that numerous barriers to care experienced by Indigenous individuals facing a life-limiting illness are addressed by a service such as an IPCNN. Future research should examine other variables measuring the impact of Indigenous palliative care navigation to develop a comprehensive understanding of the effect of this intervention. We encourage future research to assess the impact of Indigenous palliative care navigation on quality and continuity of care, end-of-life outcomes such as health care utilization and preferred place of care, as well as measures that examine the cost-effectiveness of this service.
